# Incidence of Occult Spinal Dysraphism Among Infants With Cutaneous Stigmata and Proportion Managed With Neurosurgery: A Systematic Review and Meta-analysis

**DOI:** 10.1001/jamanetworkopen.2020.7221

**Published:** 2020-07-02

**Authors:** Se Jin Choi, Hee Mang Yoon, Ji Sun Hwang, Chong Hyun Suh, Ah Young Jung, Young Ah Cho, Jin Seong Lee

**Affiliations:** 1 Research Institute of Radiology, Department of Radiology, Asan Medical Center, University of Ulsan College of Medicine, Seoul, South Korea; 2 Department of Radiology, Hallym University Medical Center, Dongtan Sacred Heart Hospital, Hwaseong, South Korea

## Abstract

**Importance:**

Occult spinal dysraphism (OSD) is the most common congenital spinal anomaly. Cutaneous anomalies such as skin dimples or deviated gluteal folds are well known as stigmata of OSD and are indicators for further evaluation; however, the association between cutaneous anomalies and OSD has not been systemically evaluated.

**Objective:**

To evaluate the incidence of OSD and the proportion of OSD cases managed with a neurosurgical intervention among neonates or infants with various cutaneous stigmata.

**Data Sources:**

PubMed and Embase databases were searched for studies published up to July 25, 2018, that evaluated the proportion of OSD cases in neonates or infants with cutaneous stigmata. Search terms included *ultrasound, dysraphism, dimple*, and *infant *or* neonate*. The search was limited to English-language publications.

**Study Selection:**

Two reviewers selected the studies evaluating the incidence of OSD among neonates or infants with cutaneous stigmata.

**Data Extraction and Synthesis:**

The Preferred Reporting Items for Systematic Reviews and Meta-analyses (PRISMA) guidelines for data extraction were followed. Pooled proportions of OSD cases and OSD cases that were managed with a neurosurgical intervention were obtained using the generalized linear mixed model and maximum likelihood method.

**Main Outcome and Measures:**

The pooled incidence of OSD and OSD cases managed with neurological surgery among patients with cutaneous stigmata was the primary outcome. This outcome was also evaluated in each subgroup, and heterogeneity was explored using subgroup analysis.

**Results:**

A total of 15 studies, involving 6558 neonate or infant patients with various cutaneous stigmata, were included. The pooled proportion of OSD cases among the patients with cutaneous stigmata was 2.8% (95% CI, 2.1%-3.8%; *I*^2^ = 51.6%), and the proportion managed with neurological surgery was 0.6% (95% CI, 0.3%-1.3%; *I*^2^ = 66.4%). Cases with combined stigmata showed a significantly higher association with OSD than those with a single stigma (10.5% [95% CI, 6.9%-15.8%] vs 2.3% [%, 95% CI, 1.5%-3.5%]; *P* < .001). The pooled proportion of OSD cases among patients with an atypical dimple was significantly higher than among those with simple dimple (8.8% [95% CI, 4.5%-16.6%] vs 0.6% [95% CI of 1.4%-2.1%]; *P* = .001).

**Conclusions and Relevance:**

The proportion of OSD in healthy, asymptomatic patients with midline cutaneous stigmata was low, and the proportion of patients who underwent a neurosurgical intervention was even lower. However, a careful evaluation as well as potential spinal magnetic resonance imaging is recommended for neonates or infants with combined stigmata or an atypical dimple for possible high-risk lesions.

## Key Points

**Question:**

What is the incidence of occult spinal dysraphism (OSD) in neonates or infants with various cutaneous stigmata, and how many of these cases are managed with a neurosurgical intervention?

**Findings:**

In this systematic review and meta-analysis of 15 studies involving 6558 patients, the pooled proportion of OSD among cases with cutaneous stigmata was 2.8%, and in 0.6% of patients, the condition was managed with neurological surgery. A stronger association with OSD was found in patients with combined stigmata and atypical dimple.

**Meaning:**

The findings of this study suggest that although the risk of OSD and the proportion of cases managed by neurosurgery were low, the characteristics of any cutaneous stigmata should be carefully evaluated in neonates or infants with midline cutaneous stigmata.

## Teaser

This systematic review and meta-analysis evaluates the incidence of occult spinal dysraphism and the proportion of cases managed with a neurosurgical intervention among neonates or infants with various cutaneous stigmata.

## Introduction

Occult spinal dysraphism (OSD) refers to a broad spectrum of skin-covered congenital spinal anomalies, including midline mesenchymal, neural, and bony elements. It is caused by an incomplete closure of the neural tube and anomalous development of the caudal cell mass during embryogenesis.^1^The clinical spectrum of OSD is broad, ranging from skin anomalies to motor, urinary, or bowel dysfunctions. Notably however, symptoms related to OSD are often not clinically obvious at birth and are usually subsequently revealed by a radiographic or physical examination. Therefore, affected patients present with delayed neurologic, urologic, and orthopedic symptoms and may have irreversible impairment.^2,3^

Midline cutaneous lesions have long been recognized as stigmata of OSD and as indicators for conducting spinal ultrasonography in neonates. Approximately 50% to 80% of patients with OSD have cutaneous lesions.^4^ The identification of such cutaneous anomalies is crucial for the detection of OSD and enables early intervention and prevention of irreversible neurologic deterioration or infection.

There have been multiple studies that evaluated the association between various kinds of midline cutaneous lesions and OSD.^5,6,7,8,9,10,11,12,13,14,15,16,17,18,19^ However, to our knowledge, the association between cutaneous anomalies and OSD has not been systemically evaluated. Therefore, the purpose of our current study was to evaluate the incidence of OSD in neonates and infants with various cutaneous stigmata as well as the proportion of these cases that were managed by neurosurgery.

## Methods

### Literature Search Strategy

This study was conducted and written in accordance with the Preferred Reporting Items for Systematic Reviews and Meta-analyses (PRISMA) reporting guideline.^20^ A computerized search of the PubMed and Embase databases was performed to identify studies that evaluated the frequency of OSD in neonates or infants with cutaneous stigmata. The cutoff date was July 25, 2018. The search terms used were ultraso* AND *dysraphism* OR *dimple* AND infant* OR neonate*. The bibliographies of these articles were also checked to identify other relevant articles. Our search was limited to publications in English.

### Inclusion and Exclusion Criteria

Studies or subsets of studies that evaluated the proportion of OSD cases among neonates or infants with cutaneous stigmata were included if they matched all the following criteria: (1) neonates or infants with cutaneous lesions who underwent spinal sonography as an initial screening test for the evaluation of OSD; (2) studies that contained data on at least 10 consecutive patients; (3) observational studies; and (4) studies with data on the incidence of OSD. Studies were excluded if they met any of the following parameters: (1) a case report or case series with a small number of patients (ie, <10); (2) editorials, letters, comments, review articles, or conference abstracts; (3) studies providing insufficient data on the incidence of OSD; (4) studies of patients presenting with more than cutaneous lesions (eg, congenital anomalies); and (5) studies with overlapping data and patients. Two reviewers (H.M.Y., with 6 years experience in pediatric radiology, and S.J.C., with 3 years experience in radiology) independently evaluated the eligibility of the studies for inclusion.

### Data Extraction

Data were extracted from the included studies using a standardized form. We compiled information on the following elements of study details: (1) study characteristics (authors, year of publication, duration of patient recruitment, affiliation, country of origin, study design), (2) demographic characteristics (sample size, male to female patient ratio, patient age at initial spinal sonography, and study population), and (3) use of spinal magnetic resonance imaging (MRI) as a reference standard. The following data on cutaneous stigmata was also extracted: (1) type of cutaneous stigmata, categorized into 3 groups (low risk [ie, simple dimple or deviated gluteal fold], intermediate risk [ie, vascular discoloration with or without low-risk stigmata], and high risk [ie, atypical dimple, hypertrichosis, pedunculated skin tag, fibroma pendulum, and any midline mass including lipoma]), and (2) number of cutaneous stigmata (ie, single vs combined). Finally, we extracted the following data on clinical outcomes: (1) number of patients who had any abnormal ultrasonography findings; (2) definition of tethered cord, low-lying conus medullaris level, and thick filum terminale; (3) number of patients with definite abnormal ultrasonography results after excluding any borderline findings (ie, borderline conus medullaris level or prominent filum terminale) or normal variations (ie, filar cyst); (4) number of patients diagnosed with OSD after excluding any borderline abnormal findings and normal variations; (5) individual types of OSD; and (6) number of patients managed with neurosurgery for OSD and their neurologic symptoms.

### Outcomes

The primary outcomes of our current meta-analysis were the incidence of OSD among neonate or infant patients with cutaneous stigmata and the frequency of neurosurgical management among these cases. The secondary outcomes were the results of subgroup analyses for the primary outcomes according to the number (ie, single vs combined) and type (ie, simple dimple vs atypical dimple and 3 risk groups) of stigmata and whether a reference standard (ie, spinal MRI) was used for every patient with abnormal findings on spinal ultrasonography or for a proportion of patients with abnormal ultrasonography findings.

### Statistical Analysis

The pooled proportions obtained from the included studies were analyzed using the generalized linear mixed model and maximum likelihood method. We used the Hartung-Knapp-Sidik-Jonkman method to derive confidence interval.^21^ Heterogeneity was assessed using the inconsistency index (*I*^2^) established by Higgins et al,^22^ which reflects the percentage of variation across studies that is caused by heterogeneity rather than chance. The values of more than 50% indicating the presence of substantial heterogeneity. To address possible publication bias, a funnel plot was visually evaluated and the Egger test which is a test for asymmetry of the funnel plot was used for the statistical assessment.^23^ The meta package in R version 3.6.2 (R Project for Statistical Computing) was used. Statistical significance was set at *P* <.05, and all tests were 2-tailed.

## Results

### Characteristics of the Included Studies

The process of literature search is summarized in the eAppendix in the Supplement, and the Figure illustrates the selection process. The characteristics of the 15 included studies are listed in Table 1, and the institutions at which the studies were conducted are detailed in eTable 1 in the Supplement. Among these publications, 6 studies were prospective^6,7,8,9,13,14^ and 9 were retrospective.^5,10,11,12,15,16,17,18,19^ The number of patients who underwent spinal ultrasonography screening ranged from 16 to 3884. The reported mean age of the patients at the time of ultrasonography examination ranged from 1.77 to 52.8 days but was not available for 8 studies.^5,7,12,13,14,15,16,17^ The main indications for an ultrasonogram evaluation were a low-risk stigmata in 3 studies,^5,7,8^ an intermediate-risk stigmata in 3 studies^9,15,18^ and low- to high-risk stigmata in 9 studies.^6,10,11,12,13,14,16,17,19^ Regarding a reference standard for the imaging diagnosis of OSD, the proportion of patients undergoing spinal MRI ranged from 0.4% to 31.3%.^5,6,7,8,9,10,11,12,13,16,17,19^ Spinal MRI was not performed in 1 study.^18^ All patients with an abnormal finding on ultrasonography underwent spinal MRI in 7 studies,^6,7,8,9,10,13,14^ while with a portion of patients with an abnormal finding on ultrasonography underwent MRI in 6 studies.^11,12,15,16,17,19^

**Figure.**
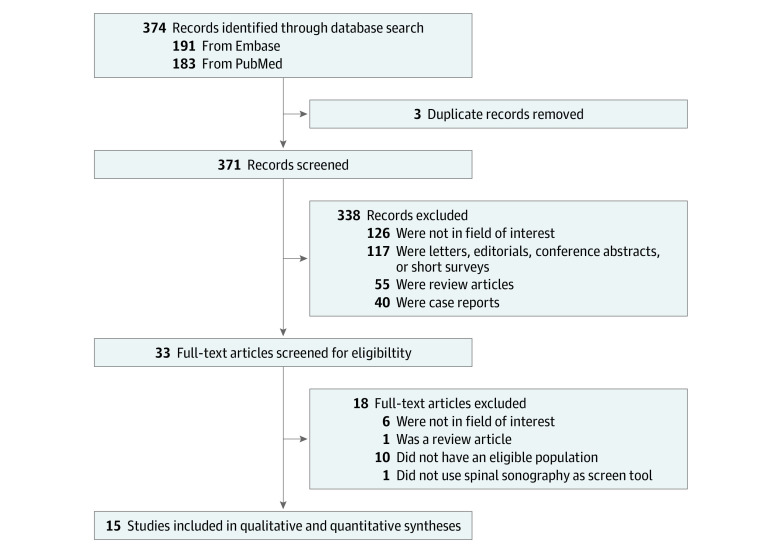
Flow Diagram of Study Selection

**Table 1.** Characteristics of the Included Studies

Source	Duration of patient recruitment	Study design	Patients undergoing spinal ultrasonography, No.	Male to female patient ratio	Age at ultrasonography evaluation, mean (range)	Study population
Allen et al,^5^ 2003	September 1993 to February 2002	Retrospective	16	9:11	NA	Patients with spinal strawberry nevi
Ausili et al,^6^ 2018	January 2012 to December 2015	Prospective	439	233:206	7 (3 to 28) d	Patients with sacral cutaneous stigmata or sacral dimple
Ben-Amitai et al,^7^ 2000	NA	Prospective	25	NA	NA	Patients with sacral nevus flammeus simplex
Ben-Sira et al,^8^ 2017	2005 to 2010	Prospective	100	36:64	8 wk (1 wk to 3 mo)	Patients with dorsal midline discoloration
Ben-Sira et al,^9^ 2009	2005 to 2007	Prospective	151	NA	7 wk (NA)	Patients with simple dimple and deviated gluteal fold
Chern et al, 2012	January 2005 to December 2009	Retrospective	943	NA	44.9 d (NA)	Patients with cutaneous stigmata
Choi et al,^11^ 2018	March 2014 to February 2017	Retrospective	230	130:100	52.8 (1 to 175) d^a^	Patients with sacral dimple
Gibson et al,^12^ 1995	NA	Retrospective	94	NA	NA	Patients with sacral hollow
Henriques et al,^13^ 2005	NA	Prospective	144	78:66	NA (1 to 2 d)	Patients with cutaneous minor stigmas
Kriss et al,^14^ 1998	July 1993 to December 1996	Prospective	207	NA	NA	Patients with dorsal cutaneous stigmata
Kucera et al,^15^ 2015	September 2000 to August 2010^b^ and January 2000 to July 2012^c^	Retrospective	3884	NA	NA	Patients with simple sacral dimple
McGovern et al,^16^ 2013	2005 to 2011	Retrospective	216	NA	NA	Patients with sacral dimple and other cutaneous stigmata
Robinson et al,^17^ 2005	June 1990 to July 2000	Retrospective	115	NA	NA (birth date to 41 wk)	Patients with cutaneous marker
Sneineh et al,^18^ 2002	NA	Retrospective	50	26:24	32 (1 to 151) d	Patients with sacral skin dimple
Wilson et al,^19^ 2016	August 2008 to December 2014	Retrospective	151	74:77	1.77 (0 to 7) d	Patients with sacral dimple

Abbreviation: NA, not available.

^a^ Age at initial visit.

^b^ Performed at Nationwide Children’s Hospital.

^c^ Performed at Cincinnati Children’s Hospital Medical Center.

### Meta-analytic Pooled Incidence of OSD and Pooled Proportion of Patients Managed With Neurosurgery

The pooled clinical outcomes for the 15 included studies are summarized in Table 2. A total of 6558 patients examined in the included study series underwent spinal ultrasonography, among whom 426 (6.5%) had abnormal sonographic results. These abnormal results included borderline findings and normal variations, such as borderline conus medullaris level, prominent filum terminale, or filar cyst.^6,8,9,10,11^ The pooled incidence of any abnormal finding, including borderline findings and normal variations, was 5.3% (95% CI, 2.6%-10.5%). Definitions of low-lying conus medullaris and thickness of filum terminale as well as detailed information regarding excluded data of borderline results and normal variants are summarized in eTable 2 in the Supplement. The commonly used criteria for the level of low-lying conus medullaris and thickness of filum terminale were below the L2-3 intervertebral disc space and 2 mm, respectively. The definition of tethered cord was missing or vague in 11 of 15 studies (eTable 2 in the Supplement). After excluding these borderline results and normal variants, a total of 229 of 5615 patients showed a definite abnormal result on spinal ultrasonography. The pooled incidence of sonographic abnormalities was 3.1% (95% CI, 1.6%-6.0%). In 14 of 15 studies, 164 patients were diagnosed with OSD by ultrasonography and/or follow-up MRI, and the overall pooled incidence was 2.8% (95% CI, 2.4%-3.8%; *I*^2^ = 51.6%). Three studies did not report on the specific types of OSD,^10,14,19^ and the details of 128 OSD cases in the 11 other studies are described in Table 3. We found that 26 of 6364 patients underwent neurologic surgery, a pooled proportion of 0.6% (95% CI, 0.3%-1.3%; *I*^2^ = 66.4%). The relevant forest plots and funnel plots are shown in eFigures 1-5 in the Supplement. The preoperative symptoms of these cases were described in only 3 studies,^7,15,19^ and these neurologic symptoms included unremarkable (5 patients) and intermittent urinary and fecal incontinence (1 patient).

**Table 2.** Summary of the Meta-analytic Pooled Proportions for Various Clinical Outcomes Among the Included Studies

Outcome	Studies, No.	Summary estimate			*P* value for reporting bias^c^
		Pooled proportion, % (95% CI)	*P* value for heterogeneity^a^	*I*^2^, %^b^	
Abnormal findings on ultrasound					
Any	14	5.3 (2.6-10.5)	<.001	97.5	.84
Definite^5,6,7,8,9,11,12,13,15,16,17,18,19^	13	3.1 (1.6-6.0)	<.001	91.6	.89
OSD among total patients^5,6,7,8,9,10,11,12,13,15,16,17,18,19^	14	2.8 (2.1-3.8)	.006	51.6	.40
Surgical intervention among patients diagnosed with OSD^5,6,7,8,10,11,12,15,16,17,18,19^	11	16.5 (11.5-23.1)	.57	37.8	.22
Surgical intervention among total patients^5,6,7,8,9,10,11,12,15,16,17,18,19^	12	0.6 (0.3-1.3)	<.001	66.4	.67

Abbreviation: OSD, occult spinal dysraphism.

^a^ The *P* value was determined by the Q method to test the heterogeneity of the pooled data, with *P* < .05 indicating substantial heterogeneity.

^b^ Higgins index for heterogeneity (>50% indicates significant heterogeneity).

^c^ The *P* values were evaluated to assess any publication or reporting bias using Egger test. A *P* < .10 indicates significant bias. This *P* value is available when there are at least 10 included studies.

**Table 3.** Types of OSD Reported Among the Included Studies

Type of OSD	Patients with OSD, No. (%) (n = 128)
Low-lying conus medullaris or tethered cord	56 (43.8)
Low-lying conus medullaris or tethered cord with fatty filum	3 (2.3)
Tethered cord with lipoma or intraspinal lipoma	5 (3.9)
Pathologic filum terminale	26 (20.3)
Thick filum terminale	6 (4.7)
Fatty filum	20 (15.6)
Dermal sinus	11 (8.6)
Spinal lipoma or intradural lipoma	7 (5.5)
Diastematomyelia or split cord malformation	4 (3.1)
Occult spina bifida or bone dysraphism	7 (5.5)
Terminal myelocistocele	2 (1.6)
Lateral meningocele	2 (1.6)
Decreased motion	2 (1.6)
Extramedullary dorsal fluid collection	1 (0.8)
Arachnoid cyst associated with pathologic filum terminale	1 (0.8)
Arachnoid cyst	1 (0.8)

Abbreviation: OSD, occult spinal dysraphism.

Compared with the other studies (range, 0%-5.3%), 1 study reported a relatively high incidence of OSD (2 of 16 patients [12.5%]).^5^ The study was performed in patients with strawberry nevus.

### Multiple Subgroup Analysis

In a head-to-head comparison of 7 studies,^5,6,8,13,16,17,18^ the incidence of OSD was significantly higher among neonates and infants with combined stigmata (10.5%; 95% CI, 6.9%-15.8%, *I*^2^ = 38.4%) than among those with single stigmata (2.3%, 95% CI, 1.5%-3.5%, *I*^2^ = 18.8%) (*P* < .001) (Table 4). Compared with patients with an atypical dimple, those with a simple dimple showed a relatively low rate of OSD (8.8% [95% CI, 4.5%-16.6%] vs 0.6% [95% CI of 1.4%-2.1%]; *P* = .001).

**Table 4.** Pooled Incidence of Occult Spinal Dysraphism in Neonates and Infants With Various Cutaneous Stigmata According to Cutaneous Stigmata^a^

Cutaneous stigmata	Studies, No.	Summary estimate			*P* value
		Pooled proportion, % (95% CI)	*P* value for heterogeneity^b^	*I*^2^, %^c^	
Patients with stigmata					
Single^5,6,8,13,16,17,18^	7	2.3 (1.5-3.5)	.83	18.8	<.001
Combined^5,6,8,13,16,17,18^	7	10.5 (6.9-15.8)	.43	38.4	
Patients with a dimple					
Simple^6,9,12,13,14,15,16,17,18^	9	0.6 (1.4-2.1)	>.99	61.5	.001
Atypical^6,12,13,14^	4	8.8 (4.5-16.6)	.35	47.1	

^a^ Because of the small number of included studies (ie, <10), a *P* value for a reporting bias was not available.

^b^ *P* value was determined by the Q method to test the heterogeneity of the pooled data, with *P* < .05 indicating substantial heterogeneity.

^c^ Higgins index for heterogeneity (>50% indicating significant heterogeneity).

Regarding the risk of cutaneous stigmata, 3 studies examined cases of low-risk stigmata,^9,15,18^ and a further 3 reports included patients with intermediate-risk stigmata, with or without a low-risk lesion.^5,7,8^ There was no study that included only patients with high-risk stigmata. There was no statistically significant difference between the pooled OSD incidence in studies of intermediate-risk stigmata cases (2.8%; 95% CI, 1.1%-7.3%) compared with those of low-risk stigmata cases (0.6%; 95% CI, 0.2%-14.5%) (*P* = .36). The pooled rate of neurosurgery was significantly higher in studies that included intermediate-risk stigmata cases (2.1%; 95% CI, 0.2%-16.3%) compared with those that included low-risk stigmata cases (0.1%; 95% CI, 0.1%-0.3%) (*P* = .02) (eTable 2 in the Supplement).

We classified our studies in 2 groups according to whether all patients with abnormal sonographic findings underwent a spinal MRI. There was no statistically significant difference between the incidence of OSD in the 6 studies in which all patients with abnormal sonographic findings underwent MRI (2.7%; 95% CI, 1.4%-5.2%) compared with that in 6 other studies in which spinal MRI had not been routinely used (2.2%; 95% CI, 1.8%-2.7%) (*P* = .96). There was also no statistically significant difference between the pooled proportion of patients managed with neurosurgery in the former subgroup of studies (0.8%; 95% CI, 0.5%-1.4%) compared with the latter subgroup (0.2%; 95% CI, 0.1%-0.4%) (*P* = .08) (eTable 2 in the Supplement). All relevant forest plots are shown in eFigures 6-9 in the Supplement.

## Discussion

Spinal ultrasonography is generally preferred as a first-line imaging test for low-risk children with suspected OSD because of its cost-effectiveness,^24^ accessibility, no requirement for sedation, and good accuracy through normally incompletely ossified posterior elements.^9^ The wide use of spinal imaging in infants with cutaneous lesions requires an investigation of the evidence base for the prevalence of OSD in this population. In addition, the aggregate data from the 15 studies, involving 6558 patients, in our current systematic review and meta-analysis have revealed a 3.1% pooled proportion of OSD among patients with definite abnormal results on spinal ultrasonography and a pooled OSD rate as low as 2.8%. In addition, few of the neonates or infants examined in these prior reports underwent a neurosurgical intervention, with a pooled proportion of 0.6%.

The prevalence of OSD was significantly higher in patients with combined stigmata (10.5%) than those with a single stigmata (2.3%). This finding is consistent with the results of previous studies that reported that a combination of 2 or more midline cutaneous lesions is more likely to be associated with OSD than a single lesion.^25,26^ Therefore, when evaluating infants with dorsal midline skin lesions, meticulous screening for skin lesions is warranted to determine whether 2 or more lesions are present, given that this indicates a higher risk of OSD. However, some studies in our current review focused on specific skin lesions, such as spinal strawberry nevi^5^ and dorsal midline vascular anomalies,^8^ that are associated with other stigmata. In 1 study performed among patients with strawberry nevus,^5^ the incidence of OSD was higher (12.5%) than that reported in other studies. Therefore, there is a possibility that the types of skin lesions, rather than simply a combination of multiple lesions, could affect OSD outcomes. A future large-scale study to further elucidate the clinical significance of the different types of specific stigmata and their associations with OSD is warranted.

The pooled incidence of OSD in the patients with an atypical dimple in our included studies was significantly higher than that in patients with a simple dimple (8.8% vs 0.6%). This result was in accordance with that of a previous study,^27^ which reported atypical dimple as a high-risk factor for OSD. It is generally accepted that further evaluations via radiologic imaging and early neurosurgical referrals are required when atypical dimples exist.^28^ The most commonly used criteria for defining simple dimples are a small size (ie, <5 mm) with a midline placement within 2.5 cm of the anus and no association of other cutaneous stigmata.^6,14,25^ An atypical dimple is larger than 5 mm and located within 25 mm of the anus.^4,17^ Other criteria for an atypical dimple include deep dimple,^12,13^ dimples located cranially to the gluteal crease or outside the midline,^29^ and multiple dimples.^28^ Therefore, clinicians involved in the referral and management of infants with a sacral dimple need to be well informed about the characteristics of atypical dimples, and careful examinations should be performed to ascertain the atypical features of such lesions in clinical practice.

In terms of the degree of OSD risk for patients with cutaneous stigmata, there was no statistically significant difference between the intermediate-risk group and the low-risk group. However, the pooled proportion of infants and neonates who underwent neurosurgery was significantly higher among patients with intermediate-risk stigmata than among those with low-risk stigmata (2.1% vs 0.1%). The risk stratifications based on lumbosacral midline cutaneous lesions might better reflect the presence of clinically significant OSD requiring surgery than a mere diagnosis of OSD. However, care must still be taken with these interpretations because there have been only a small number of studies for each risk group to date.

In our current meta-analysis, various types of OSD were shown to be associated with cutaneous stigmata. The most common type was low-lying conus medullaris (or tethered cord), followed by pathologic filum terminale and dermal sinus. Therefore, in the imaging evaluation of patients with suspected OSD, the level of the conus medullaris, motion of the conus medullaris and nerve roots, morphology of the filum terminale, and tract of dorsal dermal sinus should be carefully assessed. In the included studies, the commonly used criteria for the level of low-lying conus medullaris and thickness of filum terminale were below the L2-3 intervertebral disc space and 2 mm, respectively. Regarding the definition of tethered cord, there appeared to be no consensus among studies, and its definition was missing or vague in most studies included in this meta-analysis (11 of 15). We accepted whatever definition of low-lying conus medullaris or tethered cord provided by the authors of the included studies to determine the pooled proportion of OSD and its subtypes. This might limit our results.

We aimed to investigate the number of patients with OSD who underwent neurosurgical management and to identify the main cause of surgical treatment. Only 0.6% of patients who had cutaneous stigmata underwent neurosurgical intervention, and there was limited information about preoperative symptoms. Preoperative symptoms were described in only 6 patients, and only 1 had clinically noticeable symptoms. These results did not mean that this proportion of patients truly represented the number of patients who required neurosurgical management. There was no clear indication of surgical intervention in asymptomatic patients with apparent tethered cord as observed on imaging^30^; thus, the decision to perform surgical treatment was based on the subjective preference of surgeons and patients’ guardians. The true prevalence of neurologic impairment in patients with cutaneous stigmata who require surgical intervention should be further investigated.

When OSD is suspected on a spinal ultrasonography, a spinal MRI is regarded as the criterion-standard modality for further clarification and surgical planning. Previous studies have reported a lower sensitivity of ultrasonography compared with MRI for detecting various types of OSD, such as dermal sinus tract, thickened filum, or fatty filum.^9,31^ However, because of high costs, relatively poor availability, and the need for sedation, MRI has a more limited application in some cases. Considering this situation, we divided included studies into 2 groups, stratified by whether spinal MRI was done for every case with an abnormal ultrasonography findings. Studies in which a spinal MRI was conducted for every patient with an abnormal spinal ultrasonography finding did not have a statistically significantly higher pooled proportion of cases with a diagnosis of OSD (2.7% vs 2.2%) or cases managed with neurosurgery (0.8% vs 0.2%). This finding suggests that a spinal MRI should be given careful consideration when there are increased-risk conditions and when ultrasonography results are abnormal for an early and accurate detection of OSD and to minimize the possible neurologic impairment in affected patients.

### Limitations

This study has several potential limitations of note. First, most included studies were retrospective (9 of 15). However, most reports included a relatively large number of patients (ranging from 94 to 3884) with only 2 studies^5,18^ investigating small cohorts (16 and 50 cases). Second, a considerable heterogeneity was noted among the included studies. Thus, we performed multiple subgroup analyses. Third, we did not conduct a quality assessment of individual studies. Because of the observational nature of the included studies, there was no appropriate evaluation tool for quality assessment. To overcome these issues, we used a currently available robust methodology and followed the PRISMA guideline^20^ and the Cochrane Collaboration 2013 Handbook for Diagnostic Test Accuracy Reviews^32^ for this study.

## Conclusions

In this study, the prevalence of OSD among otherwise healthy infants and newborns with midline cutaneous stigmata was as low as 2.8%, and the cases that were managed with a neurosurgical intervention was even lower, at less than 1%, in a spinal ultrasonography screening setting. Pediatricians, neurosurgeons, dermatologists, radiologists, and other clinicians involved in the management of children with dorsal midline cutaneous stigmata should carefully evaluate whether these patients have higher-risk stigmata and then consider a spinal MRI in such cases to screen for possible OSD.

## Notes

Supplement. **eAppendix. **Supplementary Methods **eTable 1. **Institutes Where Studies Were Conducted **eTable 2. **Definitions of Tethered Cord, Low-Lying Conus Medullaris, and Thick Filum Terminale In Each Study and Data on Borderline Findings and Normal Variants on Screening Ultrasonography Excluded in Analysis of the Pooled Proportion of Definite Abnormality on Ultrasonography **eTable 3. **Multiple Subgroup Analysis for Primary Outcomes **eFigure 1. **Forest Plot and Funnel Plot of Pooled Incidence of Any Abnormal Ultrasonography Finding **eFigure 2. **Forest Plot and Funnel Plot of Pooled Incidence of Definite Abnormal Finding on Ultrasonography **eFigure 3. **Forest Plot and Funnel Plot of Pooled Incidence of Occult Spinal Dysraphism Among Total Patients **eFigure 4. **Forest Plot and Funnel Plot of Pooled Proportion of Patients Managed With Surgical Intervention Among Patients Diagnosed With Occult Spinal Dysraphism **eFigure 5. **Forest Plot and Funnel Plot of Pooled Proportion of Patients Managed With Surgical Intervention Among Total Patients **eFigure 6. **Forest Plot of Pooled Incidence of Occult Spinal Dysraphism in Neonates and Infants with Single and Combined Stigmata **eFigure 7. **Forest Plot of Pooled Incidence of Occult Spinal Dysraphism (OSD) in Neonates and Infants with Simple and Atypical Dimple **eFigure 8. **Forest Plot of Pooled Incidence of Occult Spinal Dysraphism (OSD) and Pooled Proportions of Patients Managed With Neurosurgery Among Total Patients in Studies Including Low-Risk Cutaneous Stigmata and Intermediate-Risk Stigmata **eFigure 9. **Forest Plot of Pooled Incidence of Occult Spinal Dysraphism (OSD) and Pooled Proportions of Patients Managed With Neurosurgery Among Total Patients Included in Studies that Performed MRI in All Cases With Abnormal Ultrasonography Results and Studies that Did Not Perform MRI in All Cases With Abnormal Ultrasonography Results **eReferences.**
