# Supplementary material for: Incidence of Occult Spinal Dysraphism Among Infants With Cutaneous Stigmata and Proportion Managed With Neurosurgery: A Systematic Review and Meta-analysis
Source: JAMA Netw Open. 2020 Jul 2;3(7):e207221. doi: 10.1001/jamanetworkopen.2020.7221 (PMC7333023; doi:10.1001/jamanetworkopen.2020.7221)
Supplement: Supplement. — eAppendix. Supplementary Methods eTable 1. Institutes Where Studies Were Conducted eTable 2. Definitions of Tethered Cord, Low-Lying Conus Medullaris, and Thick Filum Terminale In Each Study and Data on Borderline Findings and Normal Variants on Screening Ultrasonography Excluded in Analysis of the Pooled Proportion of Definite Abnormality on Ultrasonography eTable 3. Multiple Subgroup Analysis for Primary Outcomes eFigure 1. Forest Plot and Funnel Plot of Pooled Incidence of Any Abnormal Ultrasonography Finding eFigure 2. Forest Plot and Funnel Plot of Pooled Incidence of Definite Abnormal Finding on Ultrasonography eFigure 3. Forest Plot and Funnel Plot of Pooled Incidence of Occult Spinal Dysraphism Among Total Patients eFigure 4. Forest Plot and Funnel Plot of Pooled Proportion of Patients Managed With Surgical Intervention Among Patients Diagnosed With Occult Spinal Dysraphism eFigure 5. Forest Plot and Funnel Plot of Pooled Proportion of Patients Managed With Surgical Intervention Among Total Patients eFigure 6. Forest Plot of Pooled Incidence of Occult Spinal Dysraphism in Neonates and Infants with Single and Combined Stigmata eFigure 7. Forest Plot of Pooled Incidence of Occult Spinal Dysraphism (OSD) in Neonates and Infants with Simple and Atypical Dimple eFigure 8. Forest Plot of Pooled Incidence of Occult Spinal Dysraphism (OSD) and Pooled Proportions of Patients Managed With Neurosurgery Among Total Patients in Studies Including Low-Risk Cutaneous Stigmata and Intermediate-Risk Stigmata eFigure 9. Forest Plot of Pooled Incidence of Occult Spinal Dysraphism (OSD) and Pooled Proportions of Patients Managed With Neurosurgery Among Total Patients Included in Studies that Performed MRI in All Cases With Abnormal Ultrasonography Results and Studies that Did Not Perform MRI in All Cases With Abnormal Ultrasonography Results eReferences. [file jamanetwopen-3-e207221-s001.pdf]

Supplementary Online Content

Choi SJ, Yoon HM, Hwang JS, et al. Incidence of occult spinal dysraphism among infants with cutaneous stigmata and proportion managed with neurosurgery: a systematic review and meta-analysis. *JAMA Netw Open*. 2020;3(7):e207221. doi:10.1001/jamanetworkopen.2020.7221

eAppendix. Supplementary Methods

eTable 1. Institutes Where Studies Were Conducted

eTable 2. Definitions of Tethered Cord, Low-Lying Conus Medullaris, and Thick Filum Terminale In Each Study and Data on Borderline Findings and Normal Variants on Screening Ultrasonography Excluded in Analysis of the Pooled Proportion of Definite Abnormality on Ultrasonography

eTable 3. Multiple Subgroup Analysis for Primary Outcomes

eFigure 1. Forest Plot and Funnel Plot of Pooled Incidence of Any Abnormal Ultrasonography Finding

eFigure 2. Forest Plot and Funnel Plot of Pooled Incidence of Definite Abnormal Finding on Ultrasonography

eFigure 3. Forest Plot and Funnel Plot of Pooled Incidence of Occult Spinal Dysraphism Among Total Patients

eFigure 4. Forest Plot and Funnel Plot of Pooled Proportion of Patients Managed With Surgical Intervention Among Patients Diagnosed With Occult Spinal Dysraphism

eFigure 5. Forest Plot and Funnel Plot of Pooled Proportion of Patients Managed With Surgical Intervention Among Total Patients

eFigure 6. Forest Plot of Pooled Incidence of Occult Spinal Dysraphism in Neonates and Infants with Single and Combined Stigmata

eFigure 7. Forest Plot of Pooled Incidence of Occult Spinal Dysraphism (OSD) in Neonates and Infants with Simple and Atypical Dimple

eFigure 8. Forest Plot of Pooled Incidence of Occult Spinal Dysraphism (OSD) and Pooled Proportions of Patients Managed With Neurosurgery Among Total Patients in Studies Including Low-Risk Cutaneous Stigmata and Intermediate-Risk Stigmata

eFigure 9. Forest Plot of Pooled Incidence of Occult Spinal Dysraphism (OSD) and Pooled Proportions of Patients Managed With Neurosurgery Among Total Patients Included in Studies that Performed MRI in All Cases With Abnormal Ultrasonography Results and Studies that Did Not Perform MRI in All Cases With Abnormal Ultrasonography Results

eReferences.

This supplementary material has been provided by the authors to give readers additional information about their work.

© 2020 Choi SJ et al. *JAMA Network Open.*

eAppendix. Supplementary Methods

Literature search

A total of 374 articles were initially retrieved by the systematic search. Three duplicate studies were removed and 338 articles were further excluded after screening the title and abstracts (Fig 1). Thirty-three potentially eligible studies were therefore assessed. Upon reviewing the full-text of these publications, 18 articles were excluded for the following reasons: 1 was a review article, 6 studies were not in the field of interest, 10 included indications for spinal sonography other than cutaneous anomalies, and 2 studies did not use spinal sonography as a first line modality. There were no additional articles found following a bibliography search. A final series of 15 studies involving a total of 6558 patients was therefore included in the meta-analysis [4-18].

© 2020 Choi SJ et al. *JAMA Network Open.*

eTable 1. Institutes Where Studies Were Conducted

First author (year of publication)	Institute
Allen RM et al 2003	Oregon Health & Science University and Doernbecher Children’s Hospital, Portland
Ausili E et al 2018	Catholic University of Sacred Heart, Rome, Italy
Ben-Amitai D et al 2000	Schneider Children's Medical Center of Israel
Ben-Sira L et al 2017	Tel-Aviv Sourasky Medical Center and Sackler School of Medicine, Tel-Aviv University, Tel-Aviv Israel
Ben-Sira L et al 2009	DANA Children’s Hospital, Tel Aviv Sourasky Medical Center, Israel
Chern JJ et al 2012	Children’s Hospital in Birmingham, Alabama
Choi JH et al 2018	Chungnam National University Hospital, Chungnam National University School of Medicine
Gibson PJ et al 1995	St George’s Hospital, London, UK
Henriques JG et al 2005	Hospital das Clı´nicas of Universidade Federal de Minas Gerais
Kriss VM et al 1998	University of Kentucky Medical Center Lexington
Kucera JN et al 2015	Nationwide Children’s Hospital (Columbus, OH), Cincinnati Children’s Hospital Medical Center (Cincinnati, OH)
McGovern M et al 2013	National University of Ireland Galway, Galway, Ireland

© 2020 Choi SJ et al. *JAMA Network Open.*

Robinson AJ et al 2005	St Mary's Hospital, Manchester, UK
Sneineh AK et al 2002	Alfred I. duPont Hospital for Children, Wilmington, Delaware, U.S.A.
Wilson *P* et al 2016	North Carolina Children's Hospital

© 2020 Choi SJ et al. *JAMA Network Open.*

eTable 2. Definitions of Tethered Cord, Low-Lying Conus Medullaris, and Thick Filum Terminale In Each Study and Data on Borderline Findings and Normal Variants on Screening Ultrasonography Excluded in Analysis of the Pooled Proportion of Definite Abnormality on Ultrasonography

<table>
<tr><th>Study</th><th>Tethered cord</th><th>Low lying CM</th><th>Thick filum terminale</th><th>Borderline findings or normal variants on screening ultrasound</th><th>Comment</th></tr>
<tr><td>Allen RM et al. 2003</td><td>NA</td><td>NA</td><td>NA</td><td>NA</td><td>No borderline CM or normal variants in the OSD cases</td></tr>
<tr><td>Ausili E et al. 2018</td><td>Low lying CM and eccentric location of CM within the spinal canal, often dorsal.</td><td>Below L2-L3 (below superior endplate of L3)</td><td>2mm</td><td>Filar cyst (n=6)</td><td></td></tr>
<tr><td>Ben-Amitai D et al. 2000</td><td>NA</td><td>NA</td><td>NA</td><td>NA</td><td>No borderline CM or normal variants in the OSD cases</td></tr>
<tr><td>Ben-Sira L et al. 2017</td><td rowspan="2">1. Low-lying CM (below the L2-L3 disc)
2. Normal height of CM (L2 vertebrae and above), accompanied by any of the following: syrinx, intrathecal mass or lipoma, dermal sinus, thickened terminal filum (> 2 mm or with a bulbous appearance), or absence of cord pulsatility</td><td>Inferior to L2-L3 disc</td><td>2mm</td><td>Borderline conus medullaris level (L2-L3 intervertebral disc , n=5)</td><td></td></tr>
<tr><td>Ben-Sira L et al. 2009</td><td>Below the L2-L3 intervertebral disc</td><td>2mm</td><td>Borderline conus medullaris level (L2-L3 intervertebral disc , n=2)</td><td></td></tr>
<tr><td>Chern JJ et al. 2012</td><td>NA</td><td>Below the L2–3 interspace</td><td>2mm</td><td>Patents with filar cysts were included in abnormal US findings, but the exact number of patients were not reported. Thus, we excluded this study from the analysis.</td><td></td></tr>
</table>

© 2020 Choi SJ et al. *JAMA Network Open.*

Choi JH et al. 2018	Stretch-induced dysfunction of the caudal spinal cord and conus	Below the L2–3 disc space	2mm	Filar cyst (n=57), prominent filum terminale (n=26), borderline conus medullaris level (n=26)	
Gibson PJ et al. 1995	NA	At L3 or lower	NA	NA	No borderline CM or normal variants in the OSD cases
Henriques JG et al. 2005	NA	NA	NA	NA	No borderline CM or normal variants in the OSD cases
Kriss VM et al. 1998	NA	NA	NA	NA	Only included subgroup analysis
Kucera JN et al. 2015	NA	Below the L2–L3 disc space	2mm	NA	Normal variants were not considered abnormal.
McGovern M et al. 2013	NA	NA	NA	NA	No borderline CM or normal variants in the OSD cases
Robinson AJ et al. 2005	NA	NA	2mm	NA	No borderline CM or normal variants in the OSD cases
Sneineh AK et al. 2002	NA	NA	NA	NA	No ODS cases (all patients showed CM terminating at L2 or above L2 level)

© 2020 Choi SJ et al. *JAMA Network Open.*

Wilson P et al. 2016	NA	NA	NA	NA	Filar cysts were considered normal.

CM= conus medullaris; NA = not available

eTable 3. Multiple Subgroup Analysis for Primary Outcomes

	No. of studies	Summary estimate for OSD among total patients			*P* value		No. of studies	Summary estimate for the number of patients who required neurosurgery			*P* value
Cutaneous marker		Pooled proportion (%) (95% CI)	*P* value for heterogeneity[a]	I^2 %[b]				Pooled proportion (%) (95% CI)	*P* value for heterogeneity[a]	I^2 %[b]	
Studies including only low risk cutaneous stigmata [10,16,19]	3	0.6 (0.2-14.5)	1.00	55	0.361		3	0.1 (0.1-0.3)	1.00	0	0.020
Studies including intermediate risk stigmata [6,8,9]	3	2.8 (1.1-7.3)	0.11	39			3	2.1 (0.2-16.3)	0.62	67	
MRI as a reference standard											
Studies that performed MRI in all cases showing abnormal US results [7-11,14]	6	2.7 (1.4-5.2)	0.42	65	0.957		5[c]	0.8 (0.5-1.4)	0.55	0	0.08
Studies that did not perform MRI in all cases showing abnormal US results [12,13,16-18,20]	6	2.2 (1.8-2.7)	0.07	33			6	0.2 (0.1-0.4)	0.08	39	

[a]P value was determined by the Cochran Q method to test the heterogeneity of the pooled data, with P < .05 indicating substantial heterogeneity.

[b]Higgin's index for heterogeneity (> 50%, significant heterogeneity).

[c]Reference 14 was excluded due to unavailable data.

Due to small number of included studies (<10), a P value for a reporting bias was not available

© 2020 Choi SJ et al. *JAMA Network Open.*

eFigure 1. Forest Plot and Funnel Plot of Pooled Incidence of Any Abnormal Ultrasonography Finding

A

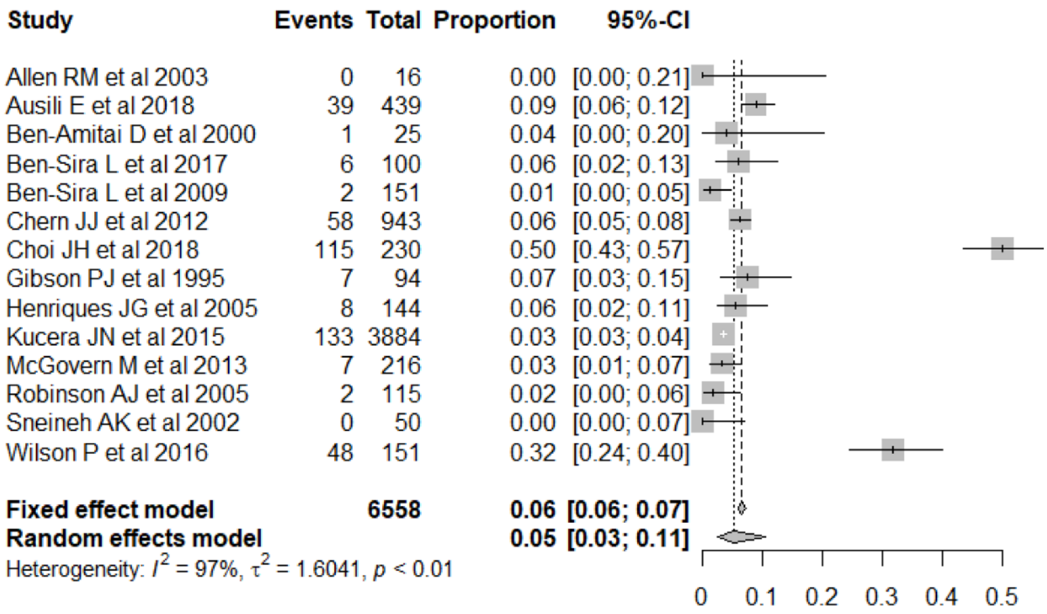

B

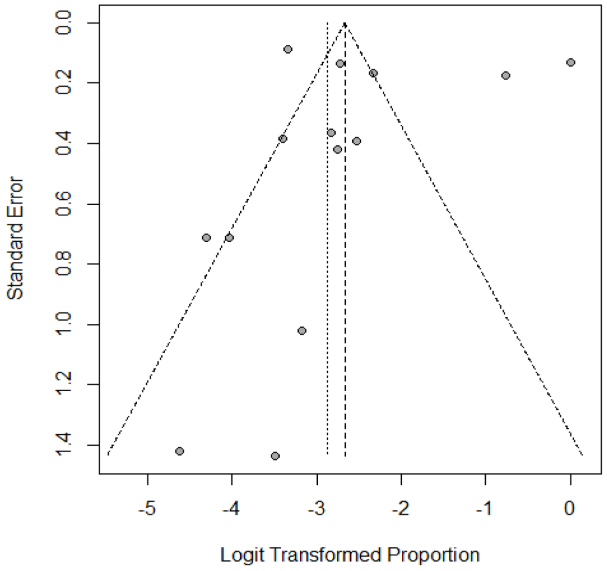

© 2020 Choi SJ et al. *JAMA Network Open.*

eFigure 2. Forest Plot and Funnel Plot of Pooled Incidence of Definite Abnormal Finding on Ultrasonography

A

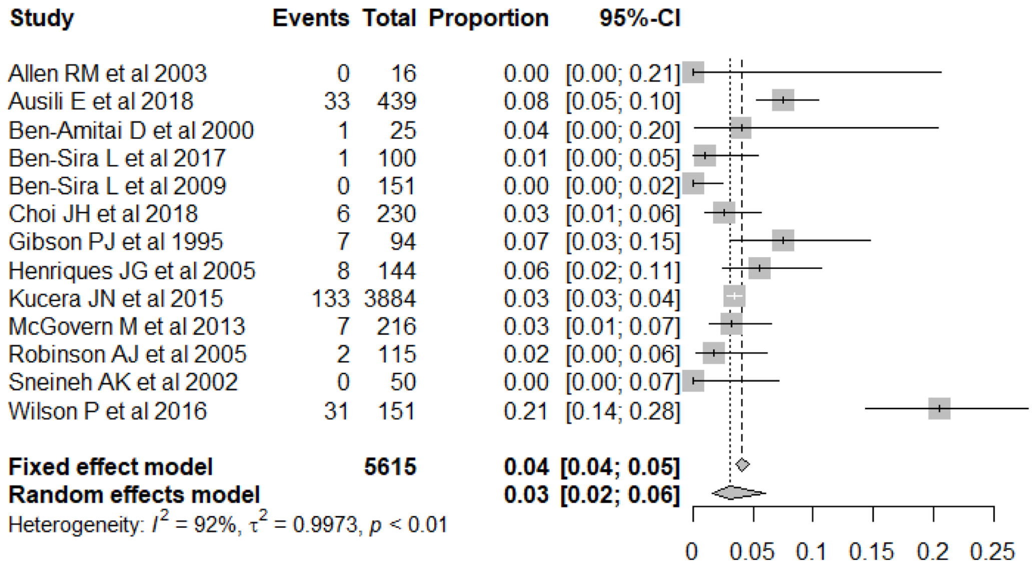

B

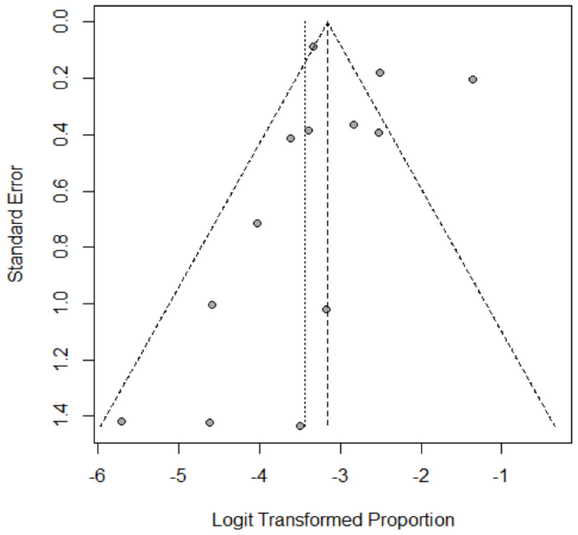

© 2020 Choi SJ et al. *JAMA Network Open.*

eFigure 3. Forest Plot and Funnel Plot of Pooled Incidence of Occult Spinal Dysraphism Among Total Patients

A

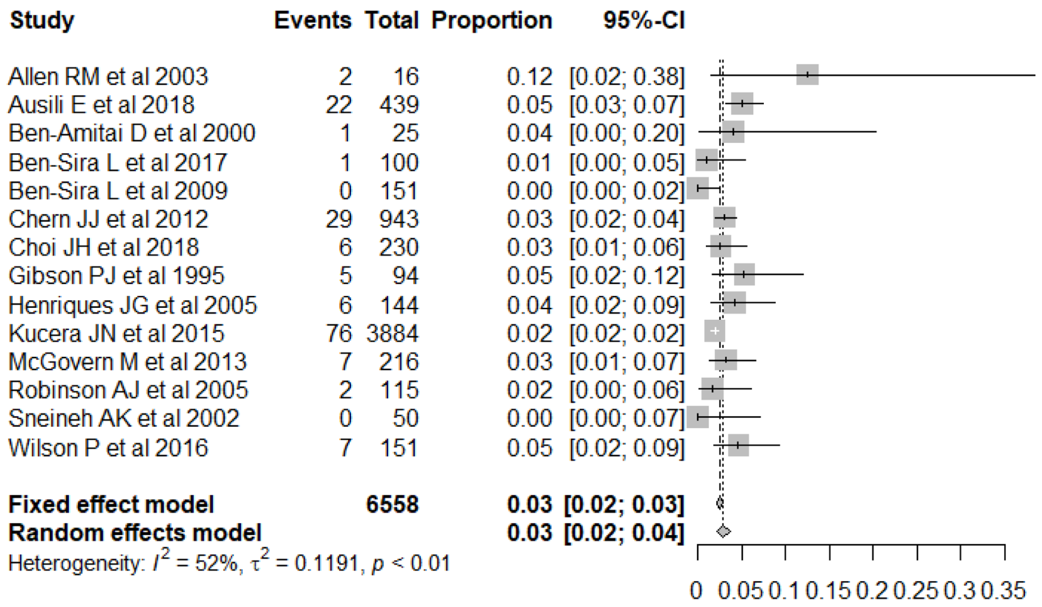

B

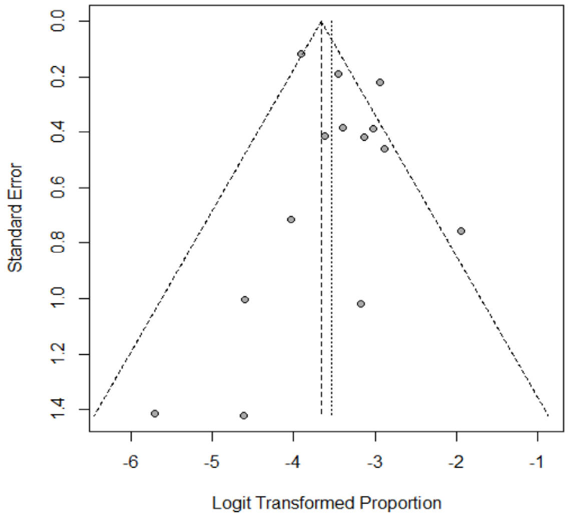

© 2020 Choi SJ et al. *JAMA Network Open.*

eFigure 4. Forest Plot and Funnel Plot of Pooled Proportion of Patients Managed With Surgical Intervention Among Patients Diagnosed With Occult Spinal Dysraphism

A

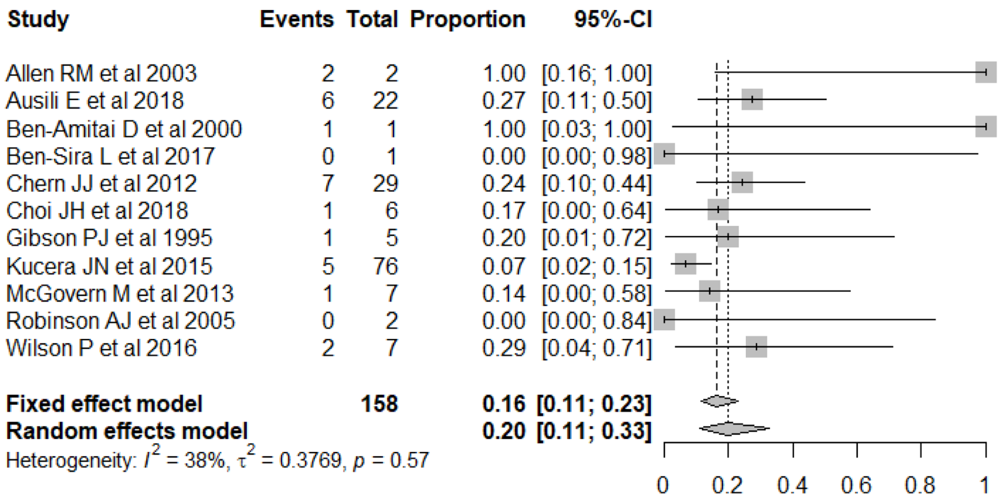

B

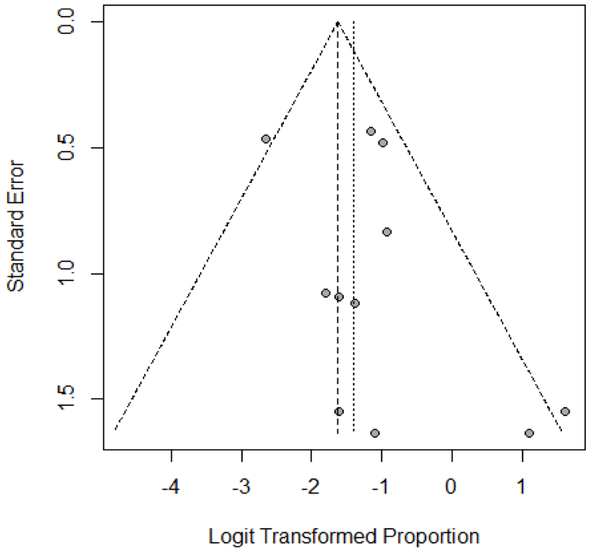

© 2020 Choi SJ et al. *JAMA Network Open.*

eFigure 5. Forest Plot and Funnel Plot of Pooled Proportion of Patients Managed With Surgical Intervention Among Total Patients

A

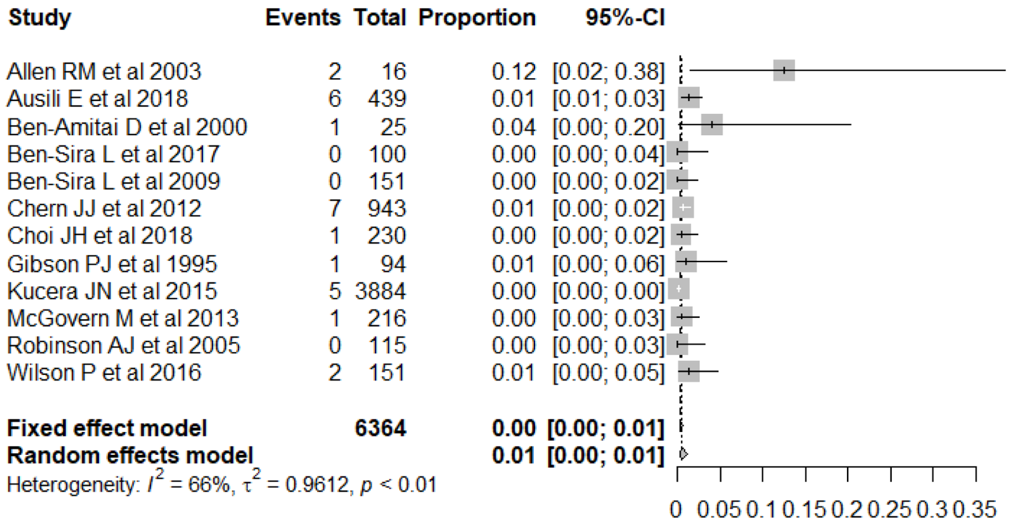

B

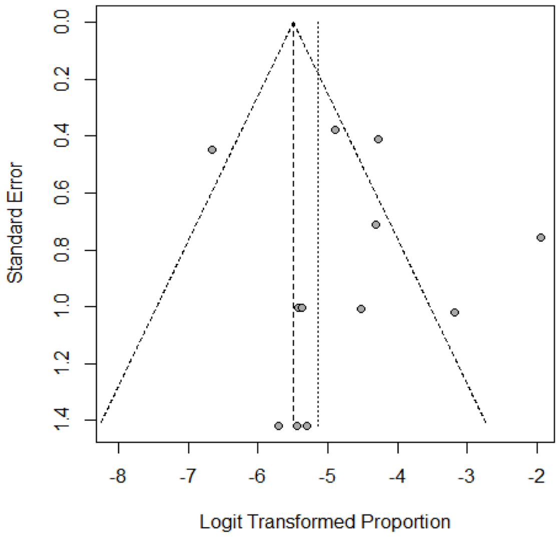

© 2020 Choi SJ et al. *JAMA Network Open.*

eFigure 6. Forest Plot of Pooled Incidence of Occult Spinal Dysraphism in Neonates and Infants with Single (A) and Combined Stigmata (B)

A

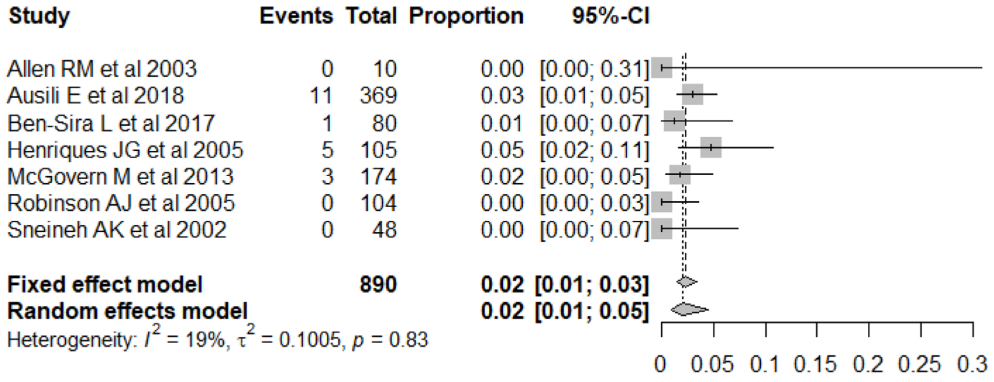

B

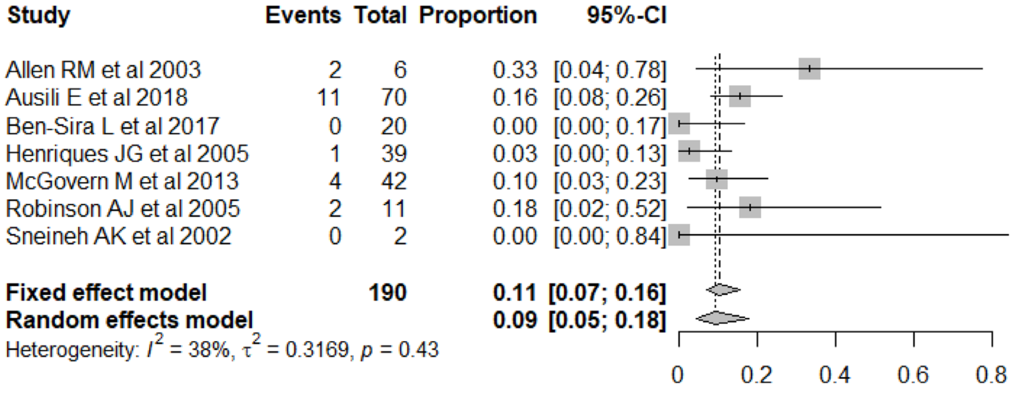

© 2020 Choi SJ et al. *JAMA Network Open.*

eFigure 7. Forest Plot of Pooled Incidence of Occult Spinal Dysraphism (OSD) in Neonates and Infants with Simple (A) and Atypical Dimple (B)

A

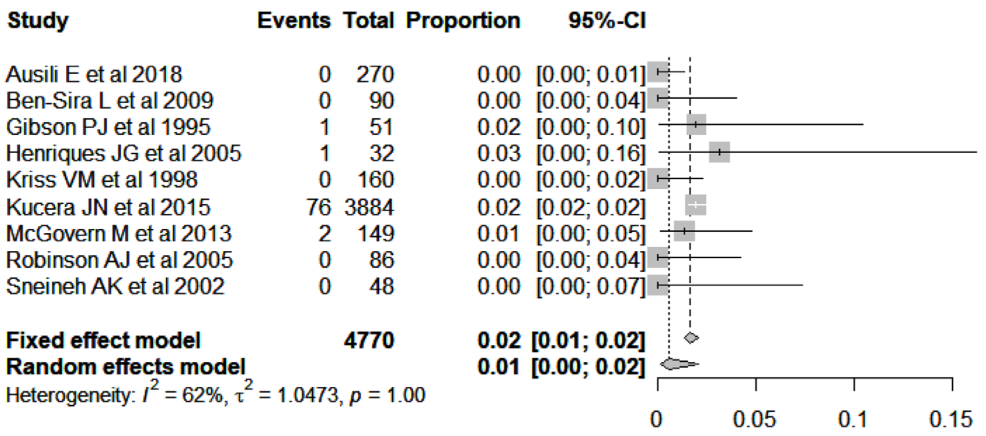

B

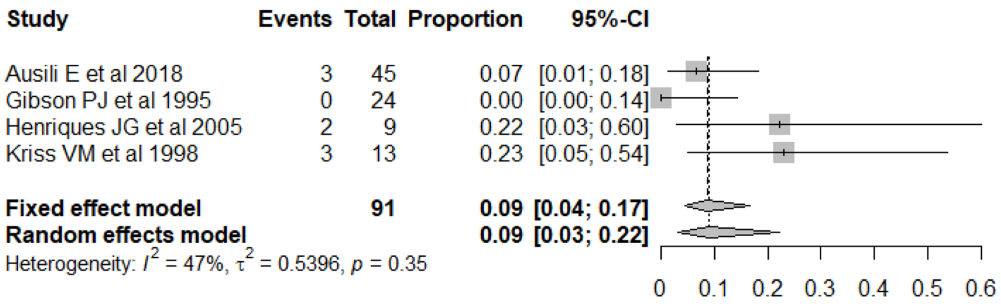

© 2020 Choi SJ et al. *JAMA Network Open.*

eFigure 8. Forest Plot of Pooled Incidence of Occult Spinal Dysraphism (OSD) (A, B) and Pooled Proportions of Patients Managed With Neurosurgery (C, D) Among Total Patients in Studies Including Low-Risk Cutaneous Stigmata (A, C) and Intermediate-Risk Stigmata (B, D)

A

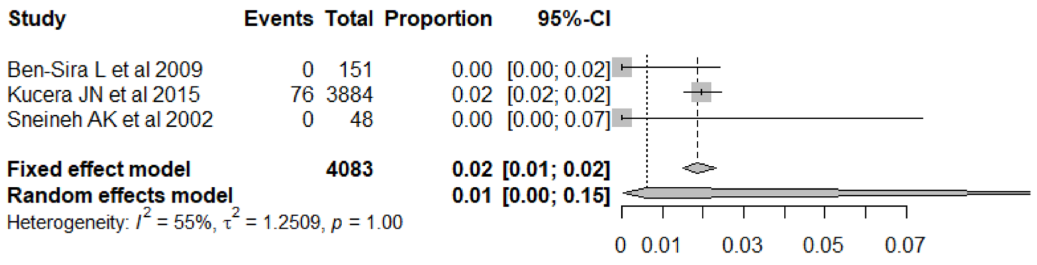

B

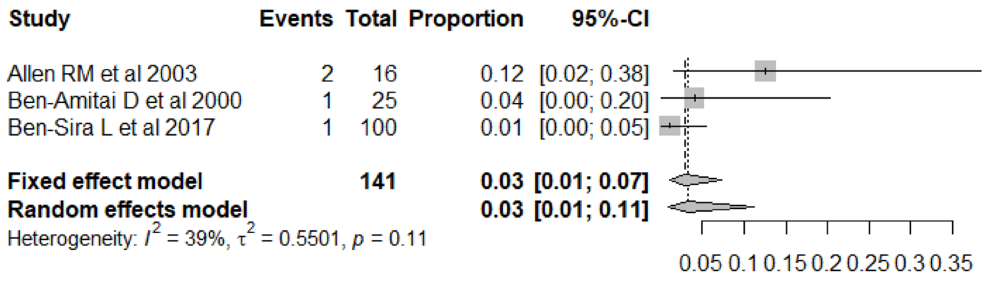

© 2020 Choi SJ et al. *JAMA Network Open.*

C

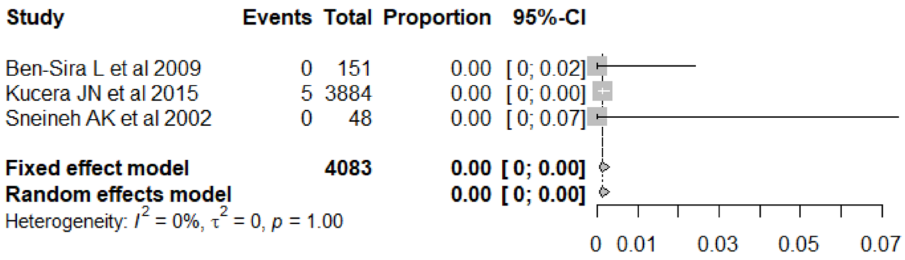

D

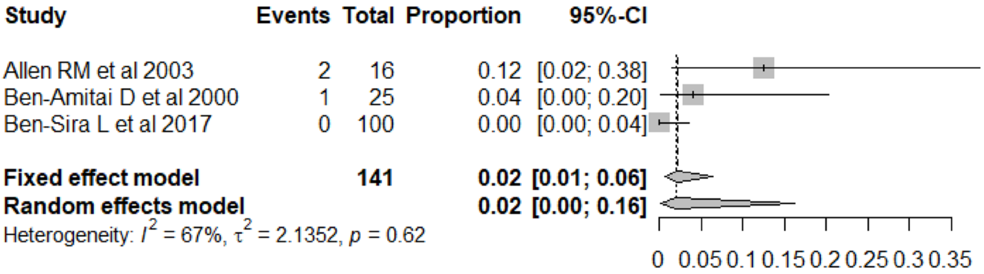

© 2020 Choi SJ et al. *JAMA Network Open.*

eFigure 9. Forest Plot of Pooled Incidence of Occult Spinal Dysraphism (OSD) (A, B) and Pooled Proportions of Patients Managed With Neurosurgery (C, D) Among Total Patients Included in Studies that Performed MRI in All Cases With Abnormal Ultrasonography Results (A, C) and Studies that Did Not Perform MRI in All Cases With Abnormal Ultrasonography Results (B, D)

A

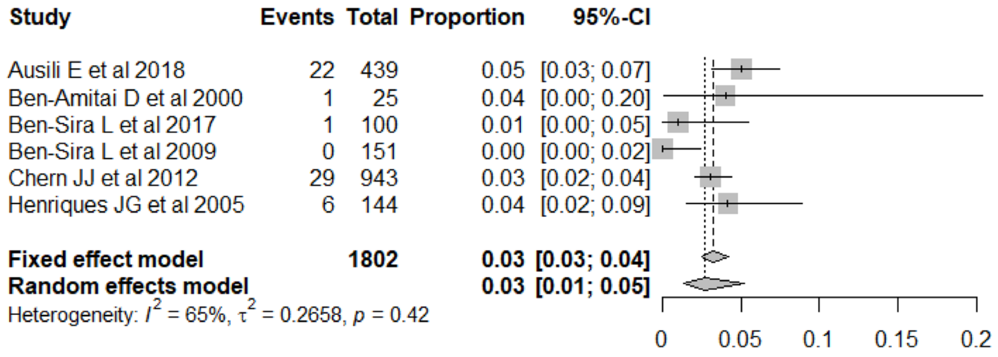

B

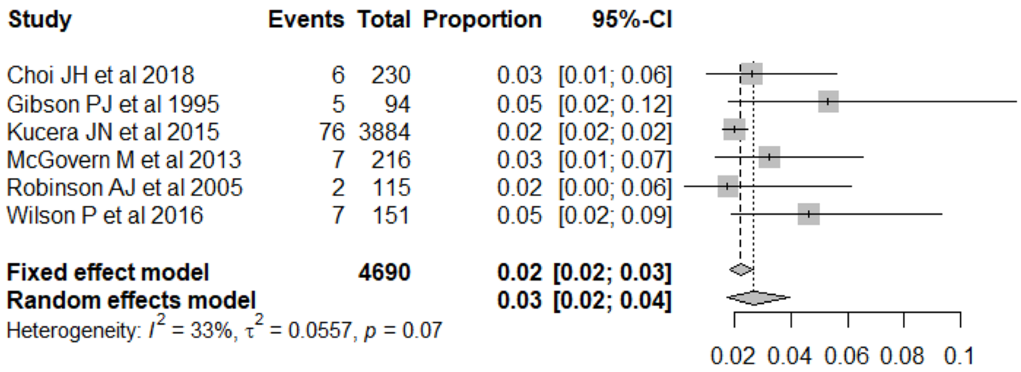

© 2020 Choi SJ et al. *JAMA Network Open.*

C

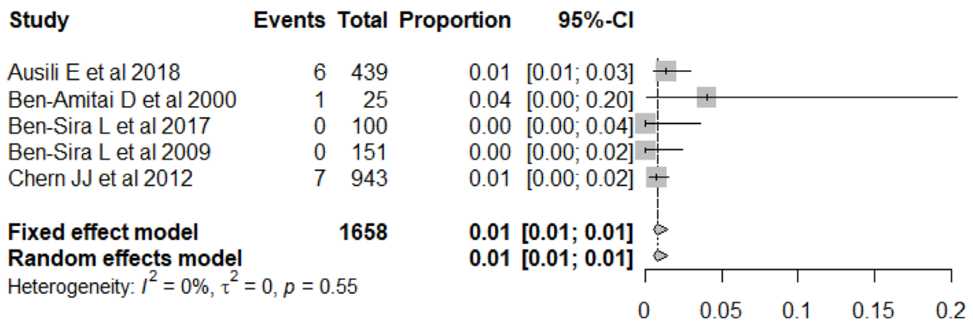

D

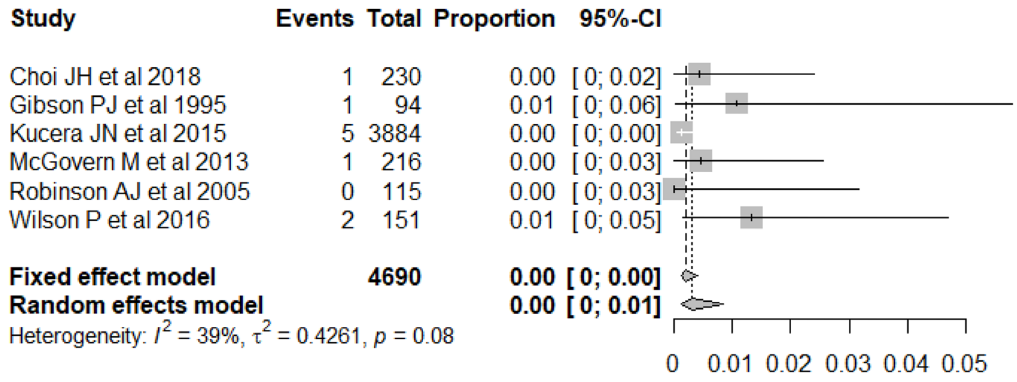

© 2020 Choi SJ et al. *JAMA Network Open.*

eReferences

1. IntHout J, Ioannidis JP, Borm GF. The Hartung-Knapp-Sidik-Jonkman method for random effects meta-analysis is straightforward and considerably outperforms the standard DerSimonian-Laird method. *BMC Med Res Methodol.* 2014;14:25. doi:10.1186/1471-2288-14-25

2. Higgins JP, Thompson SG, Deeks JJ, Altman DG. Measuring inconsistency in meta-analyses. *Bmj.* 2003;327(7414):557-560. doi:10.1136/bmj.327.7414.557

3. Egger M, Davey Smith G, Schneider M, Minder C. Bias in meta-analysis detected by a simple, graphical test. *Bmj.* 1997;315(7109):629-634. doi:10.1136/bmj.315.7109.629

4. Allen RM, Sandquist MA, Piatt JH, Jr., Selden NR. Ultrasonographic screening in infants with isolated spinal strawberry nevi. *J Neurosurg.* 2003;98(3 Suppl):247-250

5. Ausili E, Maresca G, Massimi L, Morgante L, Romagnoli C, Rendeli C. Occult spinal dysraphisms in newborns with skin markers: role of ultrasonography and magnetic resonance imaging. *Childs Nerv Syst.* 2018;34(2):285-291. doi:10.1007/s00381-017-3638-0

6. Ben-Amitai D, Davidson S, Schwartz M, et al. Sacral nevus flammeus simplex: the role of imaging. *Pediatr Dermatol.* 2000;17(6):469-471

7. Ben-Sira L, Ponger P, Constantini S. Evaluation of Dorsal Midline Discolorations with Physical Examination and Ultrasound. *J Pediatr.* 2017;190:246-250. doi:10.1016/j.jpeds.2017.07.023

8. Ben-Sira L, Ponger P, Miller E, Beni-Adani L, Constantini S. Low-risk lumbar skin stigmata in infants: the role of ultrasound screening. *J Pediatr.* 2009;155(6):864-869. doi:10.1016/j.jpeds.2009.06.003

9. Chern JJ, Kirkman JL, Shannon CN, et al. Use of lumbar ultrasonography to detect occult spinal dysraphism. *J Neurosurg Pediatr.* 2012;9(3):274-279. doi:10.3171/2011.12.peds11351

10. Choi JH, Lee T, Kwon HH, You SK, Kang JW. Outcome of ultrasonographic imaging in infants with sacral dimple. *Korean J Pediatr.* 2018;61(6):194-199. doi:10.3345/kjp.2018.61.6.194

11. Gibson PJ, Britton J, Hall DM, Hill CR. Lumbosacral skin markers and identification of occult spinal dysraphism in neonates. *Acta Paediatr.* 1995;84(2):208-209

12. Henriques JG, Pianetti G, Henriques KS, Costa P, Gusmao S. Minor skin lesions as markers of occult spinal dysraphisms--prospective study. *Surg Neurol.* 2005;63 Suppl 1:S8-12. doi:10.1016/j.surneu.2004.09.017

13. Kriss VM, Desai NS. Occult spinal dysraphism in neonates: assessment of high-risk cutaneous stigmata on sonography. *AJR Am J Roentgenol.* 1998;171(6):1687-1692. doi:10.2214/ajr.171.6.9843314

14. Kucera JN, Coley I, O'Hara S, Kosnik EJ, Coley BD. The simple sacral dimple: diagnostic yield of ultrasound in neonates. *Pediatr Radiol.* 2015;45(2):211-216. doi:10.1007/s00247-014-3110-1

15. McGovern M, Mulligan S, Carney O, Wall D, Moylett E. Ultrasound investigation of sacral dimples and other stigmata of spinal dysraphism. *Arch Dis Child.* 2013;98(10):784-786. doi:10.1136/archdischild-2012-303564

16. Robinson AJ, Russell S, Rimmer S. The value of ultrasonic examination of the lumbar spine

© 2020 Choi SJ et al. *JAMA Network Open.*

in infants with specific reference to cutaneous markers of occult spinal dysraphism. *Clin Radiol.* 2005;60(1):72-77. doi:10.1016/j.crad.2004.06.004

17. Sneineh AK, Gabos PG, Keller MS, Bowen JR. Ultrasonography of the spine in neonates and young infants with a sacral skin dimple. *J Pediatr Orthop.* 2002;22(6):761-762

18. Wilson P, Hayes E, Barber A, Lohr J. Screening for Spinal Dysraphisms in Newborns With Sacral Dimples. *Clin Pediatr (Phila).* 2016;55(11):1064-1070. doi:10.1177/0009922816664061

© 2020 Choi SJ et al. *JAMA Network Open.*
